# Sex differences in allostatic load profiles and incident dementia: The AGES-Reykjavik Study

**DOI:** 10.1177/13872877251375944

**Published:** 2025-09-09

**Authors:** Emma L Twait, Lotte Gerritsen, Vilmundur Gudnason, Lenore J Launer, Mirjam I Geerlings

**Affiliations:** 1 Department of General Practice, 1209 Amsterdam UMC, location Vrije Universiteit Amsterdam, Amsterdam, the Netherlands; 2 Amsterdam Public Health, Aging & Later life and Personalized Medicine, Amsterdam, the Netherlands; 3 Amsterdam Neuroscience, Neurodegeneration and Mood, Anxiety, Psychosis, Stress, and Sleep, Amsterdam, the Netherlands; 4 Department of Psychology, 8125 Utrecht University, Utrecht, the Netherlands; 5 Faculty of Medicine, 63541 University of Iceland, Reykjavik, Iceland; 6 64395 The Icelandic Heart Association, Kopavogur, Iceland; 7 Laboratory of Epidemiology and Population Sciences, 33251 National Institute on Aging, Baltimore, MD, USA; 8 Department of General Practice, 26066 Amsterdam UMC, location University of Amsterdam, Amsterdam, the Netherlands

**Keywords:** allostatic load, Alzheimer's disease, clustering, dementia, sex differences

## Abstract

**Background:**

Allostatic load (AL), an umbrella term for the physiological response to chronic stress, is different in women and men. AL has also been associated with all-cause dementia.

**Objective:**

The current study investigates if AL clusters differently in men and women, and if these sex-based clusters are associated with all-cause dementia.

**Methods:**

The study included individuals without dementia (n = 5343, 58% women, age range: 66–98 years) at baseline from the AGES-Reykjavik Study, a population-based cohort study. AL markers of cardiovascular, lipid, neuroendocrine, and inflammatory components were assessed at baseline. Clustering of AL markers was done using latent profile analysis in men and women separately to create sex-specific AL risk groups. Sex-specific Cox regressions on the sex-specific AL risk groups, adjusted for age, education, and medical and lifestyle factors, were performed to assess if the relationship between AL and all-cause, Alzheimer's, and non-Alzheimer's dementia differed per sex.

**Results:**

All-cause dementia was diagnosed in 1099 participants during follow-up (median: 10 years). Only cardiovascular and metabolic factors differed between AL groups in men. One of the groups in women, labeled ‘Risk factors’, was associated with a lower risk of AD dementia (HR 0.75; 95% CI 0.58; 0.98) compared to the ‘Average’ group. In men, a group labeled ‘Multisystem dysregulation’, consisting of mostly individuals with diabetes, was associated with an increased risk of all-cause dementia (HR 1.75; 95% CI 1.06; 2.90).

**Conclusions:**

AL clustered differently in men and women. Metabolic dysregulation, specifically in men, was associated with all-cause dementia.

## Introduction

Dementia, characterized by a decline in cognitive functioning, daily functioning, and quality of life, currently impacts around 50 million people worldwide and is expected to triple in number by 2050.^1^ As there currently is not a cure for dementia, focusing on modifiable risk factors can help aid in the detection of high-risk individuals as well as prevention programs. Multiple modifiable risk factors have been identified for dementia, such as cardiovascular,^2,3^ metabolic,^4,5^ lipid,^6^ inflammatory,^7,8^ and neuroendocrine^9^ factors. These biomarkers together form the umbrella term of “allostatic load” (AL), representing wear-and-tear across multiple physiological systems after repeated stressful stimuli.^10^ Assessing AL rather than individual biomarkers may give a better representation of ongoing complex disease processes.^11^

Additionally, there are significant sex differences in the epidemiology and pathophysiology of dementia.^12,13^ However, the research regarding these differences is limited and rarely focuses on AL factors. AL may emerge years before symptoms of cognitive decline and may assist the identification of high-risk individuals. In our recent study, we found four distinct AL clusters through latent profile analysis using the population-based AGES-Reykjavik Study.^14^ Further, one specific profile was associated with incident all-cause dementia, labeled ‘Multisystem dysregulation’. However, it is not known whether AL clusters the same in both men and women, and if those sex-specific clusters are associated with incident dementia. A recent meta-analysis of 21 different cohorts assessing individual cardiovascular, metabolic, and lipid factors found no sex difference in the association of these factors with dementia.^15^ There is a possibility that AL factors may interact with one another differently in men and women, and therefore should be assessed via clustering techniques rather than looking at each AL marker individually.

A recent systematic review found a discrepancy in the literature regarding sex differences and AL.^16^ While several studies found higher AL in men compared to women, a number of studies also found no differences in AL in men compared to women. One reason for this could be due to the cut-offs that are normally used to assess AL, either individually or as a sum score. These cut-offs may be sensitive to differences in sex and may explain discrepancies between previous studies, highlighting the need to either have consistent sex-specific cut-offs or assess AL on a continuous scale. Further, by defining AL through a sum score may hide possible sex-specific interactions that may exist between AL systems. Additionally, some studies have shown that women and men may show differential biological wear-and-tear in response to chronic stress.^17,18^ Differences between men and women may expand to sex differences in AL domains. Specifically, one study in the U.S. found higher immune-related AL in women and higher total, cardiovascular, and metabolic AL in men.^18^ Further research is needed on sex differences in AL, specifically by using a cluster-based approach, and to see if those sex-based clusters may explain sex disparities in dementia. Using a clustering approach takes into account the complex interactions that may exist between AL factors,^19^ and which may also differ between sexes.^20^

This study had two aims: to investigate (1) if AL clusters differently in men and women and (2) if those AL clusters differentially associate with incident dementia.

## Methods

### Participants

The current study is based on data from the Age, Gene/Environment Susceptibility (AGES)-Reykjavík Study, which is a population-based cohort study of individuals 65 years or older living in the Reykjavík area in Iceland. In short, the AGES-Reykjavík Study stems from the Reykjavík Study, which began in 1967 by the Icelandic Heart Association. A total of 5764 individuals were chosen at random from the survivors of the Reykjavík Study between 2002 and 2006. Baseline cognitive and biometric assessments were collected at the Reykjavík research center. Follow-up for incident dementia diagnosis was done until 2014. More information on the AGES-Reykjavík Study can be found elsewhere.^21^

### Standard protocol approvals, registrations, and patient consents

The Icelandic Data Protection Authority, the Icelandic National Bioethics Committee (VSN: 00-063), and the Institutional Review Board for the National Institute on Aging, NIH approved this study. Written informed consent was obtained from all participants.

### Dementia assessment

Based on international criteria, a three-step procedure was used for dementia ascertainment. Further details can be found elsewhere.^22–24^ Briefly, in step one, the total sample underwent cognitive assessment, and screen positives underwent further neuropsychological assessment. In step two, further neurologic and proxy examinations were performed on those who screened positive on neuropsychological assessments. Lastly, a multidisciplinary team of a neuropsychologist, neuroradiologist, geriatrician, and neurologist diagnosed dementia at both baseline for exclusion and at follow-up (between 2007 to 2011) based on international guidelines.^21^ Cases were also identified through medical records, nursing home records, and death certificates. For diagnoses in a nursing home, this was done for all-cause dementia and Alzheimer's disease (AD) during an intake exam and by a standardized regularly spaced exams done by all Icelandic nursing homes.^25^ The current study assessed all-cause dementia, AD, and other dementias as outcomes.

### AL measures

AL was defined based on previous research^14,26^ and separated into the following categories: cardiovascular factors, lipids, metabolic factors, inflammation, and neuroendocrine factors. Systolic blood pressure and pulse pressure were used as cardiovascular factors.^27,28^ High-density lipoprotein (HDL), low-density lipoprotein (LDL), and triglycerides were used for lipids.^26,29^ Abdominal circumference and fasting glucose were used for metabolic factors.^29,30^ High-sensitivity c-reactive protein (CRP) was used for the inflammatory factor.^31^ Morning and evening salivary cortisol were used for neuroendocrine factors.^29^ More information for how the factors were assessed can be found elsewhere.^14^

### Covariates

The covariates included age, sex, education, lifestyle, and medical variables. Age, sex, education and lifestyle variables were assessed via questionnaires at baseline. Education was divided into primary, secondary, college, or university degree. Smoking was classified as current, former, or never smoker. Alcohol use was used continuously as grams per week. Physical activity (i.e., moderate to vigorous) was categorized as never, rarely, occasionally (i.e., weekly <1 h), moderate (1–3 h per week), or high (>4 per week).^32^ Use of antidepressant or antihypertensive medication was classified as none or any. Stroke was defined through self-assessment or from hospital registries. Prevalence of *APOE* ɛ4 genotype was dichotomous and assessed via microplate array diagonal gel electrophoresis (MADGE).^33^

### Demographic information

Mild cognitive impairment was defined as scoring less than 1.5 standard deviations below a cut-point determined from the cohort on memory or two other cognitive domains^34^ and diagnosed by the same multidisciplinary panel as for dementia. Diabetes mellitus was defined as a history of diabetes, use of glucose-modifying medication, or a fasting blood glucose of more than 7 mmol/L. Metabolic syndrome was based on WHO criteria.^35,36^

### Data analysis

We excluded those with a dementia diagnosis at baseline, leaving us with 5343 individuals for the analysis.

Missing data (max: 12%) was handled using multiple imputation (10 datasets) in Mplus (v. 6.12).^14,37^ Mplus uses a Bayesian Markov chain Monte-Carlo estimation for imputation. Estimates were then pooled during the analyses.

We then assessed possible sex differences between AL factors using chi-square tests, ANOVAs, or Mann Whitney U tests. To assess interaction between profile and sex, we performed these ANOVAs on standardized AL variables to meet the assumptions of normality. We performed latent profile analysis (LPA) to determine sex-based AL profiles (i.e., stratifying by sex) using the *tidyLPA* (version 1.1.0)^38^ platform in R (version 4.2.0), which runs *MPlus*^37^ using the *MPlusAutomation* R package.^39^ Only AL factors were used to derive the sex-based profiles. To create sex-based AL profiles, LPA uses covariance across the indicator variables (i.e., the AL variables) to find relationships among individuals.^40^ We estimated 2–6 profiles to assess best model fit, based on the Akaike information criterion (AIC), the Bayesian information criterion (BIC), the sample size-adjusted BIC, and entropy. We also determined the number of profiles based on more than 1% of the sample fitting into one of the profiles. For further analyses, participants were classified based on most likely class membership.

Lastly, the sex-specific AL profiles were assessed for risk of incident all-cause dementia, AD dementia, and other dementias using Cox regressions, adjusted in a first model for age and education, and a second model additionally adjusting for lifestyle and medical variables (i.e., history of stroke, smoking, alcohol use, antidepressant and antihypertensive medication use, physical activity, and *APOE* ɛ4 genotype). The assumptions of Cox proportional hazards, influential observations, and nonlinearity were tested and met. We assessed competing risks (e.g., dementia-free mortality) as outcome as an additional sensitivity analysis. Lastly, we assessed as another sensitivity analysis an interaction between AL profiles and *APOE* ɛ4 genotype to explore if AL differs between men and women with and without an *APOE* ɛ4 genotype.

## Results

The study sample had a mean age of 77 years (age range: 66–98 years), with 58% of them being women. During a maximum of 12 years follow-up (median: 10 years follow-up), 1099 individuals were diagnosed with all-cause dementia, with 492 cases classified as AD dementia. Baseline characteristics for the total sample, as well as stratified by sex, can be found in Table 1. All allostatic load factors differed between men and women. Systolic blood pressure, abdominal circumference, evening salivary cortisol, and glucose were significantly higher in men. Whereas pulse pressure, HDL cholesterol, LDL cholesterol, morning salivary cortisol, CRP, and triglycerides were significantly higher in women.

**Table 1.** Baseline characteristics in the study sample and stratified by sex.

	Total population (n = 5343)	Men (n = 2246, 42%)	Women (n = 3097, 58%)	p
Age (y)	77 (6)	77 (6)	77 (6)	0.46
Education, college & university	1450 (27%)	726 (32%)	724 (23%)	<0.001
Current smoker	652 (12%)	260 (12%)	392 (13%)	<0.001
Alcohol use (g/week)*	3 (16)	6 (26)	2 (8)	<0.001
Physical activity, moderate/high	1694 (32%)	791 (35%)	903 (29%)	<0.001
History of stroke	348 (7%)	155 (7%)	193 (6%)	0.33
MCI at baseline	550 (10%)	275 (12%)	275 (9%)	<0.001
Metabolic syndrome	1687 (32%)	664 (30%)	1023 (33%)	0.01
Diabetes	671 (13%)	363 (16%)	308 (10%)	<0.001
Antihypertensive medication	2539 (48%)	968 (43%)	1571 (51%)	<0.001
Antidepressant medication	766 (14%)	236 (11%)	530 (17%)	<0.001
*APOE* ɛ4 genotype	1471 (28%)	647 (29%)	824 (27%)	0.07
Systolic blood pressure (mmHg)*	140 (27)	141 (26)	140 (27)	0.03
Pulse pressure (mmHg)*	67 (23)	65 (22)	68 (25)	<0.001
Abdominal circumference (cm)*	101 (15)	102 (13)	99 (17)	<0.001
HDL (mmol/L)*	1.5 (0.6)	1.4 (0.5)	1.7 (0.6)	<0.001
LDL (mmol/L)*	3.5 (1.5)	3.2 (1.4)	3.6 (1.4)	<0.001
Morning cortisol (nmol/L)*	17 (16)	16 (14)	18 (17)	0.003
Evening cortisol (nmol/L)*	2 (3)	3 (3)	2 (3)	<0.001
C-reactive protein (mg/L)*	1.9 (2.9)	1.8 (2.6)	2.0 (3.1)	0.001
Triglycerides (mmol/L)*	1.1 (0.7)	1.0 (0.7)	1.1 (0.7)	<0.001
Fasting glucose (mg/dL)*	5.5 (0.8)	5.7 (0.9)	5.5 (0.8)	<0.001

Means and standard deviations or numbers and percentages are shown, else for * indicating median and interquartile range. MCI: mild cognitive impairment; *APOE*: apolipoprotein E; HDL: high-density lipoprotein; LDL: low-density lipoprotein. Imputed values are shown for: 6% missing data on education, 1% on metabolic syndrome, 1% on systolic and diastolic blood pressure, 1% on abdominal circumference, < 1% on HDL and LDL cholesterol, 9% on morning and evening cortisol, < 1% on c-reactive protein, < 1% on triglycerides, < 1% on glucose, < 1% on *APOE* ɛ4 genotype, 3% on smoking status, 4% on alcohol use, 12% on physical activity, 2% on stroke history, and 3% on antihypertensive use.

**Figure 1.**
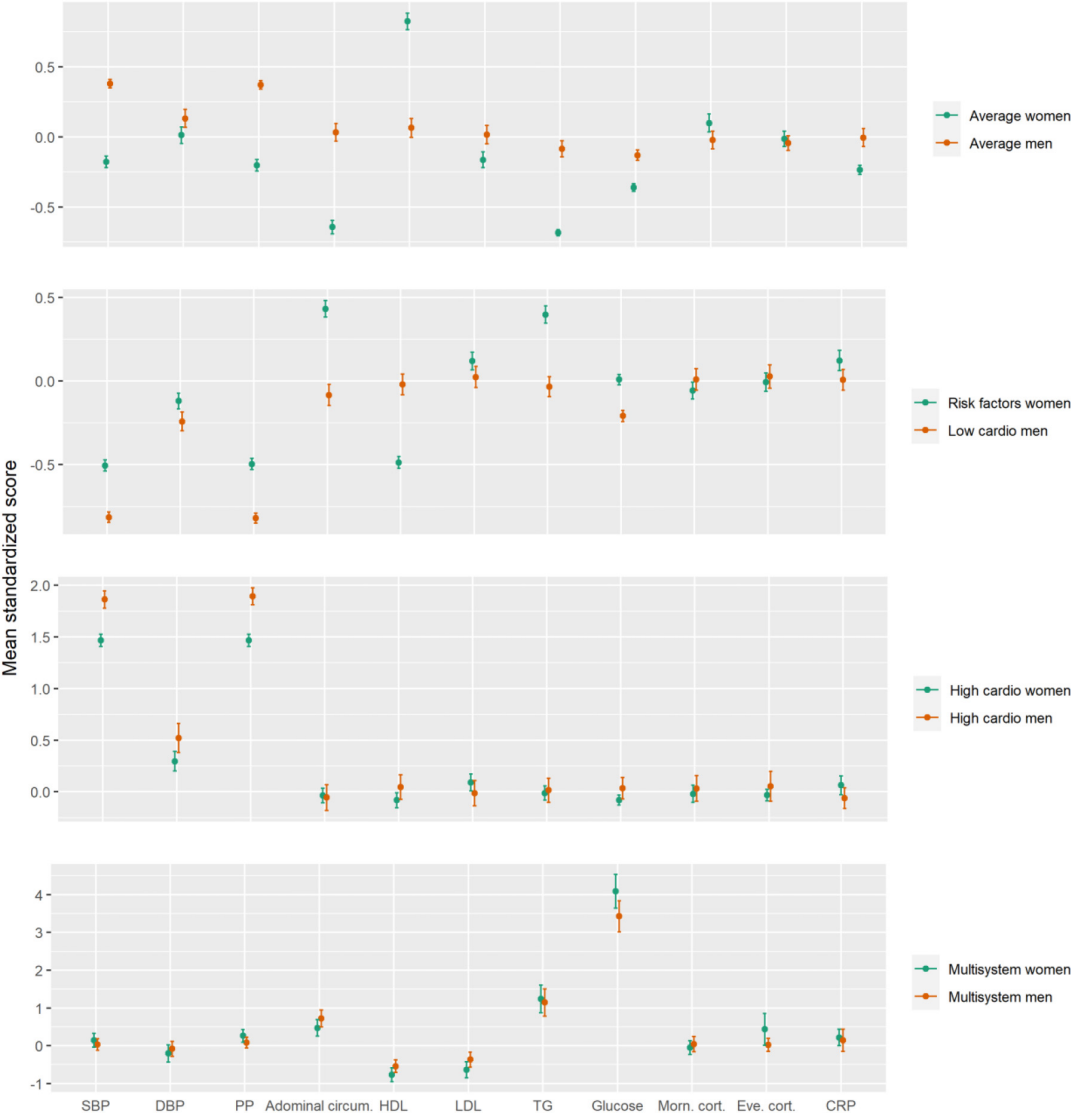
Averaged AL factor z-score and 95% confidence intervals per sex-stratified profile. SBP: systolic blood pressure; DBP: diastolic blood pressure; PP: pulse pressure; HDL: high density lipoprotein; LDL: low-density lipoprotein; TG: triglycerides; CRP: c-reactive protein. Panels are on different scales for visualization purposes.

Based on model fit, the four-class model for both men and women was chosen (see Supplemental Table 1). Entropy increased in the four-class model for both men and women, and then decreased; therefore, we chose for the smallest amount of profiles with the best fit statistics to prevent overfitting. This also allowed for direct comparison between AL profiles across sexes. Fit statistics seemed slightly better in men compared to women (Supplemental Table 1). ANOVAs revealed a significant interaction between sex and profile membership on standardized systolic blood pressure (F = 27.22, p < 0.001), pulse pressure (F = 33.64, p < 0.001), abdominal circumference (F = 37.19, p < 0.001), HDL (F = 78.32, p < 0.001), LDL (F = 5.81, p = 0.003), CRP (F = 9.22, p < 0.001), triglycerides (F = 33.18, p < 0.001), and glucose (F = 37.25, p < 0.001). However, there was no significant interaction between sex and profile membership on morning cortisol (F = 2.51, p = 0.08) or evening cortisol (F = 3.53, p = 0.01). Illustration of the averaged z-scores per AL factor, profile, and sex can be found in Figure 1. In comparison to the profiles found in the total population, larger discrepancies between them were found with the profiles found in women compared to those found in men. Standardized means and confidence intervals per AL factor and profile in the total population and in women and men separately are found in Supplemental Figure 1. Proportions of individuals above clinical cut-offs per AL factor and profile are shown in Supplemental Table 2.

### AL differences between profiles in women

In women, the profile with the highest proportion we labeled ‘Risk factors’ (46%), as this group had increased levels of risk factors (i.e., having metabolic syndrome, prevalent type 2 diabetes, taking anti-hypertensives, taking anti-depressants) compared to the ‘Average AL’ profile that had the second highest proportion (32%). The ‘Average AL’ profile was labeled as such as it had the least proportion of individuals with AL factors above their clinical cut-offs (Supplemental Table 2). The third profile we labeled ‘High cardiovascular dysregulation’ (19%), as it was characterized by high systolic blood pressure and pulse pressure (Table 2). The fourth profile we labeled ‘Multisystem dysregulation’ (3%), as it was characterized by higher levels of abdominal circumference, evening cortisol, CRP, triglycerides, and glucose. Further, lower morning cortisol was also seen in this profile (Table 3). All allostatic load factors differed between all four profiles in women.

**Table 2.** Baseline characteristics in the study sample stratified by AL profile, in women (n = 3097).

	Average AL (n = 980, 32%)	Risk factors (n = 1406, 45%)	High cardiovascular dysregulation (n = 614, 20%)	Multisystem (n = 97, 3%)	p
Age (y)	77 (6)	76 (6)	79 (6)	76 (5)	<0.001
Education, college & university	284 (29%)	299 (21%)	122 (20%)	19 (20%)	<0.001
Current smoker	132 (13%)	194 (14%)	54 (9%)	12 (12%)	0.06
Alcohol use (g/week)*	2 (10)	2 (8)	2 (8)	0 (3)	0.01
Physical activity, moderate/high	333 (34%)	384 (27%)	161 (26%)	25 (26%)	<0.001
History of stroke	43 (4%)	99 (7%)	45 (7%)	6 (6%)	0.12
MCI at baseline	86 (9%)	127 (9%)	51 (8%)	11 (11%)	0.83
Metabolic syndrome	38 (4%)	671 (48%)	226 (37%)	88 (91%)	<0.001
Diabetes	24 (2%)	130 (9%)	59 (10%)	95 (98%)	<0.001
Antihypertensive medication	384 (39%)	728 (52%)	394 (64%)	65 (67%)	<0.001
Antidepressant medication	147 (15%)	280 (20%)	82 (13%)	21 (22%)	0.001
*APOE* ɛ4 genotype	267 (27%)	359 (26%)	177 (29%)	20 (21%)	0.15
Systolic blood pressure (mmHg)*	139 (20)	131 (20)	169 (19)	144 (25)	<0.001
Pulse pressure (mmHg)*	67 (18)	60 (19)	95 (16)	73 (23)	<0.001
Abdominal circumference (cm)*	91 (14)	104 (15)	99 (15)	106 (17)	<0.001
HDL (mmol/L)*	2.1 (0.5)	1.5 (0.4)	1.6 (0.5)	1.4 (0.5)	<0.001
LDL (mmol/L)*	3.5 (1.3)	3.8 (1.4)	3.7 (1.5)	2.9 (1.5)	<0.001
Morning cortisol (nmol/L)*	19 (17)	17 (16)	16 (17)	16 (20)	<0.001
Evening cortisol (nmol/L)*	2 (2)	2 (3)	3 (3)	3 (4)	<0.001
C-reactive protein (mg/L)*	1.2 (1.6)	2.6 (3.8)	2.2 (3.1)	3.3 (4.0)	<0.001
Triglycerides (mmol/L)*	0.8 (0.3)	1.4 (0.7)	1.1 (0.6)	1.8 (1.1)	<0.001
Fasting glucose (mg/dL)*	5.2 (0.5)	5.6 (0.8)	5.5 (0.7)	9.3 (2.3)	<0.001

Means and standard deviations or numbers and percentages are shown, else for * indicating median and interquartile range. MCI: mild cognitive impairment; *APOE*: apolipoprotein E; HDL: high-density lipoprotein; LDL: low-density lipoprotein.

**Table 3.** Associations between AL profiles in women and dementia.

	No. of cases	All-cause dementia	No. of cases	AD dementia	No. of cases	Other dementias
*Model 1*						
Average AL	229	Ref.	112	Ref.	117	Ref.
Risk factors	275	0.87 (0.73; 1.03)	120	0.75 (0.58; 0.98)	155	0.97 (0.76; 1.23)
High cardiovascular	154	0.99 (0.81; 1.22)	67	0.92 (0.67; 1.25)	87	1.10 (0.83; 1.46)
Multisystem	23	1.33 (0.87; 2.04)	12	1.32 (0.73; 2.41)	11	1.35 (0.73; 2.51)
*Model 2*						
Average AL	229	Ref.	112	Ref.	117	Ref.
Risk factors	275	0.83 (0.69; 0.99)	120	0.75 (0.58; 0.98)	155	0.91 (0.71; 1.16)
High cardiovascular	154	0.98 (0.79; 1.21)	67	0.90 (0.66; 1.23)	87	1.07 (0.80; 1.43)
Multisystem	23	1.44 (0.94; 2.23)	12	1.48 (0.81; 2.71)	11	1.41 (0.75; 2.63)

AL: allostatic load; AD: Alzheimer's disease, model 1 is adjusted for age and education, model 2 is adjusted for smoking, alcohol use, physical activity, history of a stroke, hypertension, antidepressant use, and *APOE* ɛ4 genotype.

For demographic factors, highest age was seen in the ‘High cardiovascular dysregulation’ profile, highest education in the ‘Average AL’ group, lowest alcohol use in the ‘Multisystem dysregulation’ profile, highest physical activity in the ‘Average AL’ profile, highest prevalence of metabolic syndrome in the ‘Multisystem dysregulation’ profile, highest prevalence of type 2 diabetes in the ‘Multisystem dysregulation’ profile, and highest use of antidepressant medication in the ‘Multisystem dysregulation’ profile. Smoking, stroke prevalence, mild cognitive impairment at baseline, and *APOE* ɛ4 genotype did not differ between the four profiles (Table 2).

### AL differences between profiles in men

The profile in men with the highest prevalence (44%) we labeled ‘Low cardiovascular dysregulation’, as it was characterized by low systolic blood pressure and low pulse pressure compared to the other profiles (Table 4). The profile with the second highest prevalence (41%) we labeled ‘Average AL’, as it was characterized by average values across all AL variables (Supplemental Figure 2, Table 3). The third profile we labeled ‘High cardiovascular dysregulation’ (11%), as it was characterized by high systolic blood pressure and pulse pressure. Lastly, there was a profile we labeled ‘Multisystem dysregulation’ (4%) that was characterized by higher abdominal circumference, high triglycerides, and high glucose. Morning cortisol, evening cortisol, and CRP did not differ between the four profiles in men (Table 4).

**Table 4.** Baseline characteristics in the study sample stratified by AL profile, in men (n = 2246).

	Average AL (n = 898, 40%)	Low cardiovascular (n = 1010, 45%)	High cardiovascular dysregulation (n = 246, 11%)	Multisystem (n = 92, 4%)	p
Age (years)	77 (5)	76 (6)	79 (6)	76 (6)	<0.001
Education, college & university	295 (33%)	326 (32%)	75 (30%)	30 (33%)	0.03
Current smoker	94 (10%)	133 (13%)	21 (9%)	12 (13%)	0.01
Alcohol use (g/week)*	6 (26)	6 (26)	10 (27)	6 (25)	0.05
Physical activity, moderate/high	317 (35%)	360 (36%)	82 (33%)	32 (35%)	0.26
History of stroke	67 (7%)	66 (7%)	14 (6%)	8 (9%)	0.81
MCI at baseline	116 (13%)	114 (11%)	33 (13%)	12 (13%)	0.78
Metabolic syndrome	259 (29%)	247 (24%)	81 (33%)	77 (84%)	<0.001
Diabetes	122 (14%)	98 (10%)	51 (21%)	92 (100%)	<0.001
Antihypertensive medication	419 (47%)	325 (32%)	171 (70%)	53 (58%)	<0.001
Antidepressant medication	100 (11%)	105 (10%)	19 (8%)	12 (13%)	0.49
*APOE* ɛ4 genotype	280 (31%)	287 (28%)	54 (22%)	27 (29%)	0.05
Systolic blood pressure (mmHg)*	150 (13)	128 (14)	178 (17)	143 (22)	<0.001
Pulse pressure (mmHg)*	73 (12)	54 (11)	98 (12)	66 (19)	<0.001
Abdominal circumference (cm)*	102 (13)	101 (13)	101 (14)	109 (16)	<0.001
HDL (mmol/L)*	1.4 (0.5)	1.4 (0.5)	1.4 (0.5)	1.1 (0.4)	<0.001
LDL (mmol/L)*	3.3 (1.4)	3.3 (1.3)	3.1 (1.4)	3.0 (1.4)	0.01
Morning cortisol (nmol/L)*	16 (13)	16 (15)	17 (15)	18 (17)	0.77
Evening cortisol (nmol/L)*	3 (3)	3 (3)	3 (3)	3 (3)	0.25
C-reactive protein (mg/L)*	1.8 (2.6)	1.8 (2.6)	1.9 (2.5)	1.8 (2.5)	0.92
Triglycerides (mmol/L)*	1.0 (0.6)	1.0 (0.6)	1.1 (0.7)	1.7 (1.2)	<0.001
Fasting glucose (mg/dL)*	5.7 (0.8)	5.6 (0.7)	5.8 (0.9)	9.6 (2.6)	<0.001

Means and standard deviations or numbers and percentages are shown, else for * indicating median and interquartile range. MCI: mild cognitive impairment; *APOE*: apolipoprotein E; HDL: high-density lipoprotein; LDL: low-density lipoprotein.

With regards to demographics, age was highest in the ‘High cardiovascular dysregulation’ profile, education levels were highest in the ‘Multisystem dysregulation’ profile, smoking levels were highest in the ‘Multisystem dysregulation’ profile, metabolic syndrome and diabetes mellitus type 2 prevalence were highest in the ‘Multisystem dysregulation’ profile, and anti-hypertensive use was highest in the ‘High cardiovascular dysregulation’ profile. Alcohol use, physical activity, stroke prevalence, mild cognitive impairment at baseline, antidepressant medication use, and *APOE* ɛ4 genotype did not differ between the four groups (Table 4).

### Sex-specific AL profiles on dementia risk

For the AL profiles created among women, only the ‘Risk Factors’ group was associated with a lower risk of dementia, only in the fully-adjusted model (HR 0.83; 95% CI 0.69; 0.99, p = 0.04). For AD dementias, the ‘Risk Factors’ profile was associated with a decreased risk of AD dementias (HR 0.75; 95% CI 0.58; 0.98, p = 0.03) and remained after further adjustment for covariates (HR 0.75; 95% CI 0.58; 0.98, p = 0.03). For other dementias, no profiles were associated with increased or decreased risk, even after further adjustment for covariates (Table 3).

For the AL profiles in men, the ‘Multisystem dysregulation’ profile was associated with an increased risk of all-cause dementia (HR 1.75; 95% CI 1.06; 2.90, p = 0.03), compared to the ‘Average AL’ profile. After the adjustment of multiple confounders, the association remained (HR 1.75; 95% CI 1.06; 2.90, p = 0.03). No other profiles were associated with all-cause dementia. No profiles were associated with AD dementia, also after further adjustment for covariates. For other dementias, the ‘Multisystem dysregulation’ profile was associated with an increased risk of other dementias (HR 2.33; 95% CI 1.24; 4.38, p = 0.01), which remained after further adjustment of covariates (HR 2.23; 95% CI 1.18; 4.21, p = 0.01). No other profiles were associated with other dementias (see Table 5).

**Table 5.** Associations between AL profiles in men and dementia.

	No. of cases	All-cause dementia	No. of cases	AD dementia	No. of cases	Other dementias
*Model 1*						
Average AL	167	Ref.	77	Ref.	90	Ref.
Low cardiovascular	186	1.17 (0.95; 1.44)	84	1.17 (0.86; 1.60)	102	1.15 (0.87; 1.53)
High cardiovascular	48	0.93 (0.67; 1.28)	14	0.64 (0.36; 1.13)	34	1.18 (0.79; 1.75)
Multisystem	17	1.73 (1.05; 2.86)	6	1.34 (0.58; 3.08)	11	2.33 (1.24; 4.38)
*Model 2*						
Average AL	167	Ref.	77	Ref.	90	Ref.
Low cardiovascular	186	1.16 (0.94; 1.43)	84	1.17 (0.85; 1.60)	102	1.18 (0.88; 1.57)
High cardiovascular	48	1.01 (0.73; 1.40)	14	0.67 (0.37; 1.20)	34	1.24 (0.83; 1.85)
Multisystem	17	1.75 (1.06; 2.90)	6	1.44 (0.62; 3.34)	11	2.23 (1.18; 4.21)

AL: allostatic load; AD: Alzheimer's disease, model 1 is adjusted for age and education, model 2 is adjusted for smoking, alcohol use, physical activity, history of a stroke, hypertension, antidepressant use, and *APOE* ɛ4 genotype.

When assessing competing risks, we found an increased risk for the ‘Multisystem dysregulation’ group in both women and men for dementia-free mortality (Supplemental Table 3 and Supplemental Table 4). When looking at the interaction between AL profiles and *APOE* ɛ4 genotype, there was no evidence for interaction in women or men on AL profile and *APOE* ɛ4 genotype (Supplemental Table 5).

## Discussion

The current study aimed to explore sex differences in clustering AL as well as its relationship to incident dementia. All AL variables discriminated the clusters in women, whereas in men, only cardiovascular, lipid, and metabolic factors discriminated between the clusters. Further, in men the ‘Multisystem dysregulation’ group was associated with almost a two-times higher risk of incident all-cause dementia. When clustering AL in both men and women together, this profile was also associated with increased risk of incident dementia.^14^ However, when assessing sex-specific AL clusters, this profile was only associated with an increased risk in men, highlighting the importance of assessing sex differences in dementia. Lastly, in women, a decreased risk was seen in the ‘Risk factors’ profile, specifically for AD dementia.

A recent systematic review found that men may have higher total AL than women,^16^ but it was unclear which specific aspects of AL may drive this sex difference. The current study found dominant discrimination of endocrine and immune factors when clustering in women, whereas cardiovascular and metabolic factors were the most important factors in men during clustering. These findings are in line with a recent narrative review by Longpré-Poirier, Dougoud,^41^ that suggested these sex differences in AL as well. This highlights the need for classification of AL through clustering rather than using sum scores or predefined cut-offs when assessing sex differences.

The current study found a sex-specific effect solely of the ‘Multisystem dysregulation’ group on all-cause dementia in men only. While a previous study assessed sex differences in individual risk factors for dementia,^15^ the AL factors assessed in that study (i.e., cholesterol and abdominal circumference), that were increased in the ‘Multisystem dysregulation’ group, showed no differences in sex on incident dementia. This discrepancy could be explained by the clustering of risk factors in the current study. The clustering of AL factors into risk profiles could more accurately reflect multimorbidity and dementia risk.^42^ It is important to note that almost everyone in this group had type 2 diabetes, and the increased risk on incident dementia could also be explained by the presence of type 2 diabetes. When adding type 2 diabetes as an additional confounder to the fully-adjusted model, this indeed drove the relationship between the ‘Multisystem dysregulation’ group and incident dementia. The previous studies on sex differences regarding diabetes and dementia have thus far been mixed.^15,43,44^ A previous study^43^ found a difference between early- and late-onset of diabetes, with increased risk seen during midlife onset compared to later-life onset of type 2 diabetes. Lastly, our competing risk analysis found an increased risk of dementia-free mortality in the ‘Multisystem dysregulation’ group in women. This is in line with a previous meta-analysis showing an increased relative risk of mortality in women with type 2 diabetes compared to men.^45^ There is a possibility that women in this risk profile had a lower life expectancy, and therefore died before receiving a dementia diagnosis.

Lastly, the current study found a decreased risk of AD dementia in the ‘Risk factors’ profile in women compared to the ‘Average AL’ profile. While many women in this profile showed multiple risk factors, such as being on anti-hypertensive medication, their blood pressure was comparable to the ‘Average AL’ group. Previous literature has shown that antihypertensive medication use may be protective for dementia.^46^ There is a possibility that the anti-hypertensive medication is acting as a protective factor for incident dementia in this group. Previous literature has also found that women show a protection against the negative effects of hypertension and metabolic dysregulation, which may be explained by sex differences in the renin-angiotensin system.^47^ Further, a large majority of the group also was physically active, which could also be preventing dementia.^48^ Lastly, we speculate that this group had other cognitive resilience factors, such as certain social factors^49^ or lifelong wealth,^50^ that could have compensated for their biological risk factors and therefore reduced their overall risk. It would be of interest for future studies to assess the role of cognitive reserve when assessing sex differences of allostatic load on dementia. Previous studies have suggested that cognitive reserve may protect against allostatic load^51^ and may be further implicated by sex differences.^52^

Strengths of the study include the LPA methodology to assess AL on a continuous scale, a large, community-based cohort of approximately equal proportion of men and women to assess sex differences, as well as a long follow-up time for incident dementia assessment. Further, we used imputation to handle missing data and include multiple confounders. However, the study also came with some limitations. The subtyping of other dementias was not done in all participants, particularly those diagnosed in nursing homes; therefore, our results on AD and other dementias cannot be inferred with categorical certainty. Further, the ‘Multisystem dysregulation’ profile consisted of a small proportion of the study population, and therefore may have been underpowered. Replication studies are therefore warranted. We also did not have information on diet, which could have been driving the null association and lowered triglycerides observed^53^ in the ‘High cardiovascular’ group. Also, it is important to note that the AGES-Reykjavik Study is ethnically homogeneous. As psychological stress, AL, sex, and ethnicity have been shown to intersect, it is crucial to assess sex differences in AL in marginally underrepresented populations as well, who may be disproportionately affected by stressors.^54,55^

The current study revealed distinct patterns in AL between women and men. Future studies assessing AL should prioritize including interactions with sex. Metabolic dysregulation, specifically in men, was associated with a higher risk of all-cause dementia. These findings advance our understanding of the biological underpinnings of sex differences in dementia and highlight metabolic disruption in men as a potential target for early intervention and prevention strategies.

## Supplemental Material

sj-docx-1-alz-10.1177_13872877251375944 - Supplemental material for Sex differences in allostatic load profiles and incident dementia: The AGES-Reykjavik Study Supplemental material, sj-docx-1-alz-10.1177_13872877251375944 for Sex differences in allostatic load profiles and incident dementia: The AGES-Reykjavik Study by Emma L Twait, Lotte Gerritsen, Vilmundur Gudnason, Lenore J Launer and Mirjam I Geerlings in Journal of Alzheimer's Disease
