# Supplementary material for: Sex differences in allostatic load profiles and incident dementia: The AGES-Reykjavik Study
Source: J Alzheimers Dis. 2025 Sep 9;108(1):193–203. doi: 10.1177/13872877251375944 (PMC12541116; doi:10.1177/13872877251375944)
Supplement: sj-docx-1-alz-10.1177_13872877251375944 - Supplemental material for Sex differences in allostatic load profiles and incident dementia: The AGES-Reykjavik Study [file sj-docx-1-alz-10.1177_13872877251375944.docx]

**Supplemental Material**

**Sex differences in allostatic load profiles and incident dementia: The AGES-Reykjavik Study**


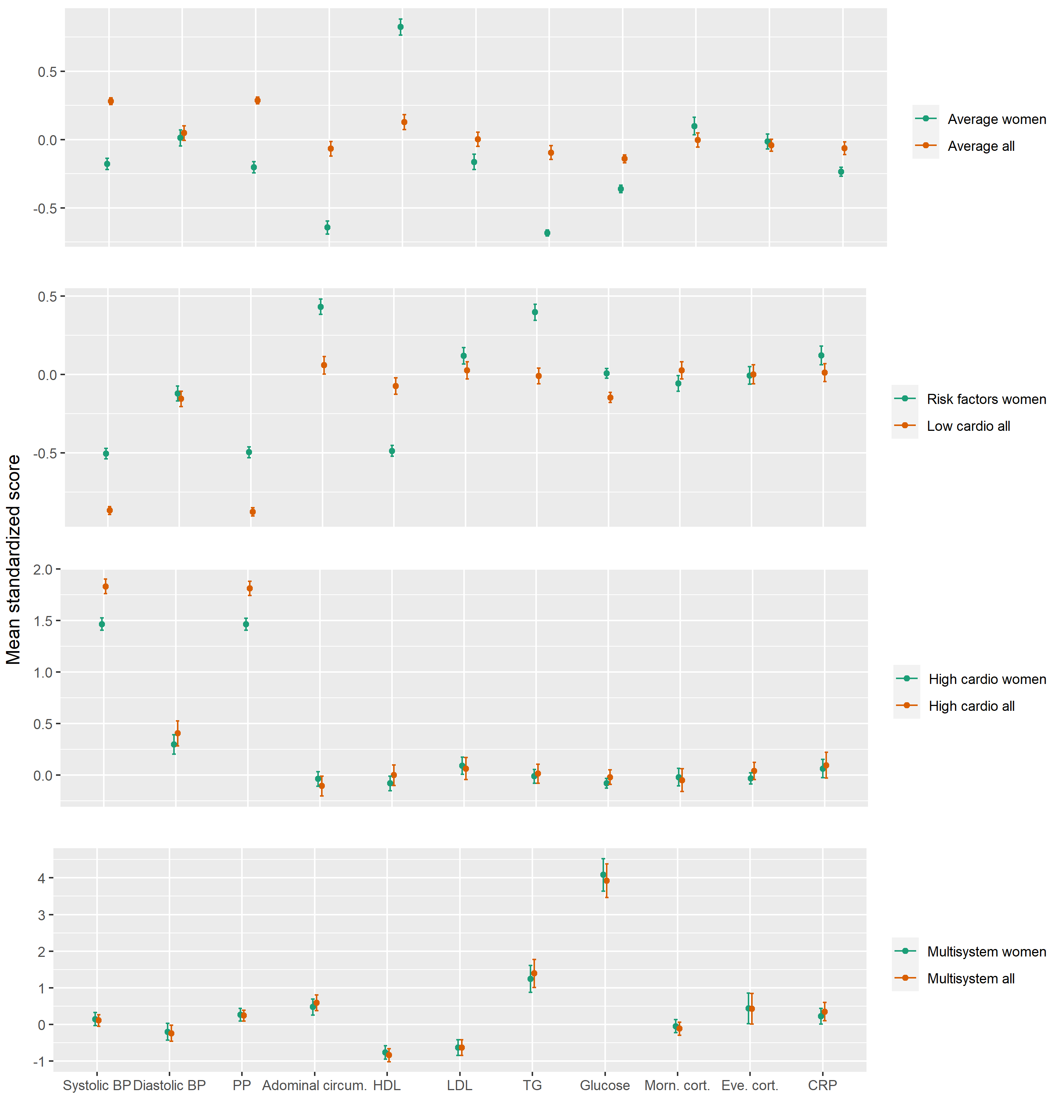


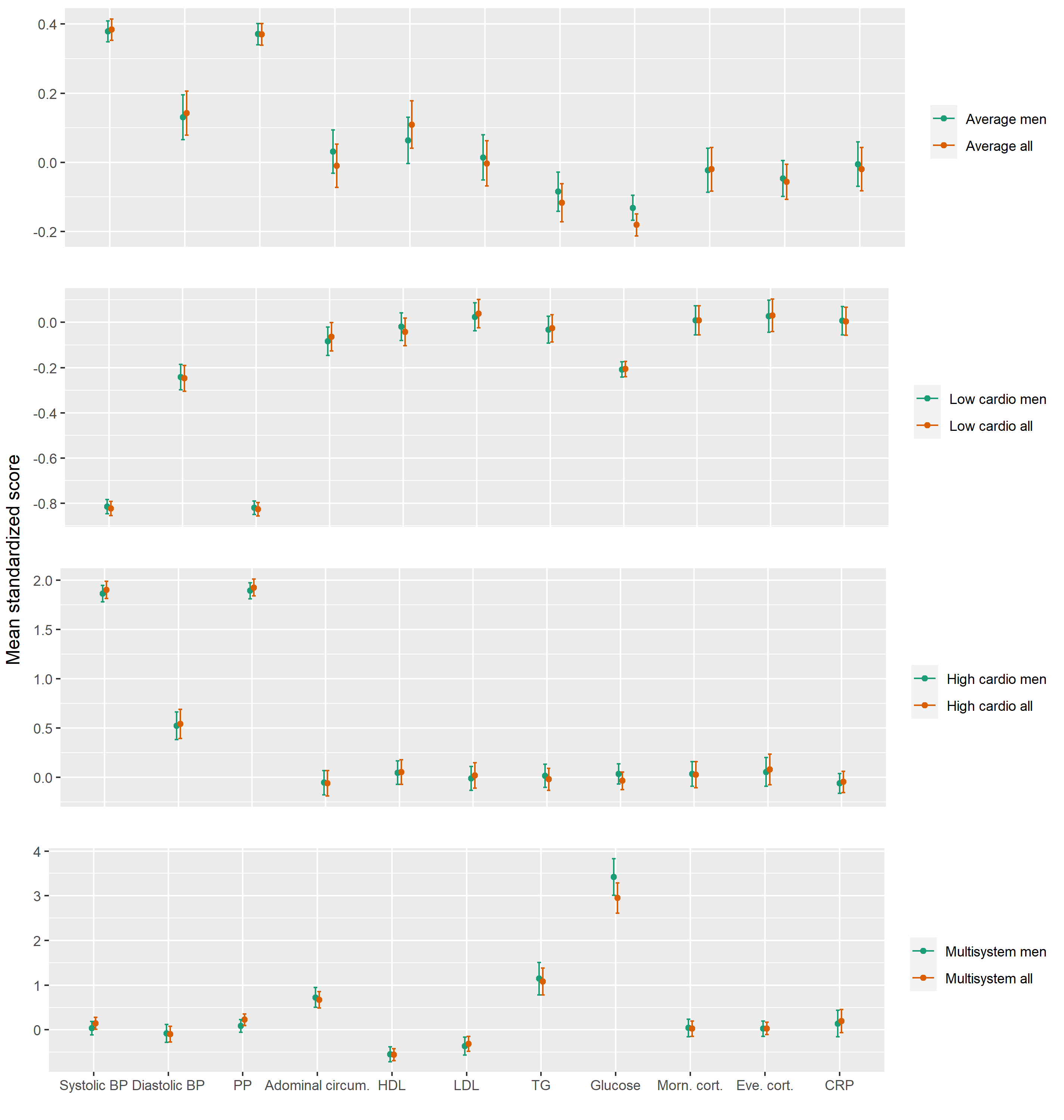


**Supplemental Figure 1.** Comparison of the standardized means and 95% confidence intervals per AL factor in the sex-stratified clusters in comparison to the previous clusters on the total population.^1^

**Supplemental Table 1.** Fit statistics of models with 2 to 6 classes for women and men.

| **Model** | **LL** | **AIC** | **BIC** | **saBIC** | **Entropy** |
| --- | --- | --- | --- | --- | --- |
| *Women* |  |  |  |  |  |
| 2 classes | -42839 | 85740 | 85927 | 85828 | 0.83 (0.77; 0.84) |
| 3 classes | -42171 | 84426 | 84680 | 84546 | 0.73 (0.73; 0.86) |
| 4 classes | -41608 | 83322 | 83642 | 83474 | 0.79 (0.79; 0.85) |
| 5 classes | -41228 | 82584 | 82971 | 82767 | 0.78 (0.78; 0.80) |
| 6 classes | -40902 | 81954 | 82407 | 82169 | 0.81 (0.79; 0.82) |
|  |  |  |  |  |  |
| *Men* |  |  |  |  |  |
| 2 classes | -31078 | 62219 | 62396 | 62298 | 0.81 (0.79; 0.83) |
| 3 classes | -30672 | 61429 | 61669 | 61535 | 0.80 (0.79; 0.87) |
| 4 classes | -30320 | 60746 | 61049 | 60881 | 0.83 (0.76; 0.84) |
| 5 classes | -30084 | 60297 | 60663 | 60459 | 0.77 (0.76; 0.79) |
| 6 classes | -29909 | 59969 | 60398 | 60159 | 0.76 (0.76; 0.79) |

LL: log-likelihood; AIC: Akaike information criterion; BIC: Bayesian information criterion; saBIC: sample size-adjusted Bayesian information criterion. Confidence intervals for entropy were calculated by using 10 bootstrapped samples (10 was selected due to computational reasons). While the confidence intervals for the 4-class model for men (0.83; 95% CI: 0.76–0.84) overlaps with the 2- and 3-class models, it is higher than the 5-class (0.77; 95% CI: 0.76–0.79) and 6-class (0.76; 95% CsI: 0.76–0.79) models. This is in line with our rationale to use a 4-class model to capture complexity without sacrificing interpretability. All confidence intervals overlapped for women, which is in line with our rationale to use a 4-class model for interpretability.

**Supplemental Table 2.** Frequencies of allostatic load clinical cut-offs per profile in women and in men.

*Women*

|  | Total | Average | Risk factors | High cardiovascular | Multisystem |
| --- | --- | --- | --- | --- | --- |
| Systolic blood pressure > 140 mmHg ^2^ | 49% | 46% | 28% | 99% | 61% |
| Diastolic blood pressure > 90 mmHg ^2^ | 3% | 2% | 1% | 6% | 3% |
| Abdominal circumference > 88 cm ^3^ | 81% | 61% | 94% | 84% | 90% |
| HDL < 1.3 mmol/L ^4^ | 16% | 1% | 25% | 16% | 43% |
| LDL > 3.4 mmol/L ^4^ | 59% | 54% | 65% | 61% | 34% |
| Morning cortisol > 13.2 nmol/L ^5^ | 66% | 70% | 65% | 63% | 62% |
| Evening cortisol > 10.1 nmol/L ^6^ | 5% | 5% | 5% | 4% | 12% |
| C-reactive protein > 3.0 mg/L ^7^ | 36% | 19% | 46% | 39% | 56% |
| Triglycerides > 2.6 mmol/L ^7^ | 4% | 0% | 6% | 3% | 25% |
| Fasting glucose > 5.6 mg/Dl ^8^ | 36% | 17% | 43% | 38% | 100% |

*Men*

|  | Total | Average | Low cardiovascular | High cardiovascular | Multisystem |
| --- | --- | --- | --- | --- | --- |
| Systolic blood pressure > 140 mmHg ^2^ | 51% | 87% | 7% | 100% | 61% |
| Diastolic blood pressure > 90 mmHg ^2^ | 7% | 8% | 3% | 19% | 4% |
| Abdominal circumference > 102 cm ^3^ | 48% | 50% | 45% | 47% | 72% |
| HDL < 1.0 mmol/L ^4^ | 12% | 10% | 11% | 14% | 25% |
| LDL > 3.4 mmol/L ^4^ | 43% | 43% | 45% | 38% | 30% |
| Morning cortisol > 13.2 nmol/L ^5^ | 64% | 62% | 64% | 66% | 65% |
| Evening cortisol > 10.1 nmol/L ^6^ | 6% | 4% | 7% | 6% | 5% |
| C-reactive protein > 3.0 mg/L ^7^ | 30% | 31% | 30% | 29% | 27% |
| Triglycerides > 2.6 mmol/L ^7^ | 4% | 3% | 3% | 2% | 19% |
| Fasting glucose > 5.6 mg/dL ^8^ | 50% | 50% | 44% | 57% | 100% |

**Supplemental Table 3.** Competing risk analysis of dementia and mortality in women.

|  | **All-cause dementia** | **All-cause mortality** | **Dementia-free mortality** | **Dementia and/or mortality** |
| --- | --- | --- | --- | --- |
| *Model 1* |  |  |  |  |
| Average | Ref. | Ref. | Ref. | Ref. |
| Risk factors | 0.88 (0.73; 1.05) | 0.92 (0.81; 1.05) | 1.05 (0.89; 1.23) | 0.97 (0.86; 1.09) |
| High cardiovascular | 1.00 (0.81; 1.23) | 1.07 (0.92; 1.24) | 1.16 (0.96; 1.41) | 1.09 (0.94; 1.25) |
| Multisystem | 1.33 (0.87; 2.04) | 1.76 (1.34; 2.33) | 2.05 (1.47; 2.85) | 1.71 (1.32; 2.22) |
|  |  |  |  |  |
| *Model 2* |  |  |  |  |
| Average | Ref. | Ref. | Ref. | Ref. |
| Risk factors | 0.84 (0.73; 0.99) | 0.89 (0.78; 1.01) | 1.02 (0.86; 1.20) | 0.93 (0.83; 1.05) |
| High cardiovascular | 1.00 (0.81; 1.21) | 1.10 (0.94; 1.28) | 1.22 (1.01; 1.48) | 1.11 (0.97; 1.28) |
| Multisystem | 1.46 (0.87; 2.23) | 1.84 (1.39; 2.44) | 2.15 (1.54; 3.01) | 1.83 (1.40; 2.38) |

**Supplemental Table 4.** Competing risk analysis of dementia and mortality in men.

|  | **All-cause dementia** | **All-cause mortality** | **Dementia-free mortality** | **Dementia and/or mortality** |
| --- | --- | --- | --- | --- |
| *Model 1* |  |  |  |  |
| Average | Ref. | Ref. | Ref. | Ref. |
| Low cardiovascular | 1.16 (0.94; 1.43) | 1.08 (0.95; 1.22) | 1.09 (0.94; 1.26) | 1.11 (0.98; 1.25) |
| High cardiovascular | 0.92 (0.67; 1.28) | 0.97 (0.81; 1.17) | 1.03 (0.83; 1.27) | 0.99 (0.83; 1.18) |
| Multisystem | 1.67 (1,00; 2.80) | 2.01 (1.54; 2.63) | 2.18 (1.61; 2.95) | 2.03 (1.56; 2.63) |
|  |  |  |  |  |
| *Model 2* |  |  |  |  |
| Average | Ref. | Ref. | Ref. | Ref. |
| Low cardiovascular | 1.15 (0.93; 1.42) | 1.09 (0.96; 1.24) | 1.11 (0.96; 1.29) | 1.12 (0.99; 1.27) |
| High cardiovascular | 1.00 (0.72; 1.39) | 1.00 (0.83; 1.21) | 1.03 (0.83; 1.29) | 1.02 (0.85; 1.22) |
| Multisystem | 1.68 (1.00; 2.83) | 1.92 (1.47; 2.52) | 2.05 (1.51; 2.78) | 1.95 (1.50; 2.53) |

**Supplemental Table 5.** Sensitivity analysis on the interaction between AL profiles and *APOE* ε4 genotype.

|  | **All-cause dementia** |
| --- | --- |
| *Interaction in women* |  |
| Average * *APOE* ε4 genotype | Ref. |
| Risk factors * *APOE* ε4 genotype | 0.86 (0.59; 1.24) |
| High cardiovascular * *APOE* ε4 genotype | 0.82 (0.54; 1.24) |
| Multisystem * APOE ε4 genotype | 0.75 (0.26; 2.13) |
|  |  |
| *Interaction in men* |  |
| Average * *APOE* ε4 genotype | Ref. |
| Low cardiovascular * *APOE* ε4 genotype | 0.68 (0.44; 1.05) |
| High cardiovascular * *APOE* ε4 genotype | 0.94 (0.48; 1.85) |
| Multisystem * *APO*E ε4 genotype | 1.01 (0.34; 2.97) |

Corrected for age, education, smoking, alcohol use, physical activity, history of a stroke, hypertension, and antidepressant use.

**References**

1. Twait EL, Basten M, Gerritsen L, et al. Late-life depression, allostatic load, and risk of dementia: The AGES-Reykjavik study. *Psychoneuroendocrinology* 2023; 148: 105975.

2. Chobanian AV, Bakris GL, Black HR, et al. Seventh report of the Joint National Committee on Prevention, Detection, Evaluation, and Treatment of High Blood Pressure. *Hypertension* 2003; 42: 1206-1252.

3. Ross R, Neeland IJ, Yamashita S, et al. Waist circumference as a vital sign in clinical practice: a Consensus Statement from the IAS and ICCR Working Group on Visceral Obesity. *Nat Rev Endocrinol* 2020; 16: 177-189.

4. Lee Y and Siddiqui WJ. Cholesterol levels. *StatPearls*. Treasure Island (FL): StatPearls Publishing, 2025.

5. Kim YJ, Kim JH, Hong AR, et al. Stimulated salivary cortisol as a noninvasive diagnostic tool for adrenal insufficiency. *Endocrinol Metab (Seoul)* 2020; 35: 628-635.

6. van Baal L, Wichert M, Zwanziger D, et al. Distinct late-night salivary cortisol cut-off values for the diagnosis of hypercortisolism. *Horm Metab Res* 2021; 53: 662-671.

7. Bruun-Rasmussen NE, Napolitano G, Bojesen SE, et al. Correlation between allostatic load index and cumulative mortality: a register-based study of Danish municipalities. *BMJ Open* 2024; 14: e075697.

8. ElSayed NA, Aleppo G, Aroda VR, et al. 2. Classification and diagnosis of diabetes: standards of care in diabetes—2023. *Diabetes Care* 2022; 46: S19-S40.
